# The Scientific American

**Published:** 1894-12

**Authors:** 


THE SCIENTIFIC AMERICAN

Is probably the best known scientific journal in the world. It is also quite an old journal, having been established in 1845. Coming out every week, it contains the latest news of a scientific character in every department of knowledge.

The number for November 17th is particularly interesting to physicians because of the elaborate article it contains upon the new method of treating croup and diphtheria, entitled "Serum-therapy."

The article is made more interesting by the illustrations representing the method of injecting the serum into the patient; of withdrawing the therapeutic serum from the horse, and of the preparation of the toxin.

There is in addition the usual miscellaneous scientific information that appeals to men of every pursuit.

